# Clinical characterization and outcome of prolonged heart rate-corrected QT interval among children with syndactyly

**DOI:** 10.1097/MD.0000000000022740

**Published:** 2020-10-16

**Authors:** Hao Han, Youzhou Chen, Songnan Li, Lan Ren, Jianqiang Zhang, Huayi Sun, Jianzeng Dong, Xingshan Zhao

**Affiliations:** a Department of Cardiology, Beijing Jishuitan Hosptial, No. 31 East Street, Xinjiekou, XiCheng; b Department of Cardiology, Beijing Anzhen Hospital, Capital Medical University, Beijing; c Department of Cardiology, Shanghai East Hospital, Shanghai, China.

**Keywords:** electrocardiography, long QT syndrome, pediatrics, syndactyly, timothy syndrome

## Abstract

Prolonged heart rate-corrected QT (QTc) interval is an independent risk factor for sudden cardiac death, which is the hallmark of Timothy syndrome (TS). There are little data on children with syndactyly and QTc prolongation.

To evaluate the characteristics and long-term outcomes in children with syndactyly, and to attempt to identify TS in patients with syndactyly and QTc prolongation.

This is a retrospective case-control study of children with syndactyly who visited Beijing Jishuitan Hospital between July 2003 and February 2013. The patients with prolonged QTc intervals are matched 1:4 with patients without prolongation. Genetic testing of the *CACNA1C* gene is routinely performed in patients with QTc prolongation.

The mean age at admission is 3.4 ± 2.3 years. Compared with the normal QTc group, those with QTc prolongation showed higher frequencies of congenital heart disease (11.8% vs 1.5%, *P* = .042), mental retardation and facial dysmorphia (11.8% vs 0, *P* = .004), and T wave alternans (23.5% vs 4.4%, *P* = .01). In the multivariable analysis, only T wave alternans (OR = 10.61, 95%CI: 1.39–81.16, *P* = .023) is independently associated with QTc prolongation in patients with syndactyly. One child with QTc prolongation had a mutation in the *CACNA1C* gene. No patients with prolonged QTs interval met the threshold for TS.

Children with syndactyly and prolonged QTc interval had more multisystem diseases and electrocardiography abnormalities. T wave alternans is independently associated with QTc prolongation in patients with syndactyly.

## 1 Introduction

Syndactyly is a common hereditary hand malformation (incidence > 0.03%^[1]^): adjacent fingers and/or toes are webbed or fused.^[2]^ Syndactyly can be inherited isolated or as part of a syndrome, like, eg, Timothy Syndrome (TS) (OMIM #601005), also referred to as syndactyly-associated long QT syndrome (LQTS).^[3]^ LQTS is characterized by prolonged ventricular repolarization, with a prolonged QT interval, which causes syncope and sudden cardiac death (SCD) from ventricular arrhythmias.^[3]^

TS is a multisystem disorder characterized by cardiac, hand/foot, facial, and neurodevelopmental features, and its diagnosis generally made within the first few days of life.^[3]^ The presence of a pathogenic variant in CACNA1C (often de novo) is proof of the diagnosis. Ventricular tachyarrhythmia is usually the leading cause of death at the age of 2.5 years on average.^[4]^ The alternatively spliced exon 8A of the *CACNA1C* gene^[5]^ results in the inactivation of the Ca_v_1.2 L-type calcium channel and in a prolonged heart rate-corrected QT (QTc) of 480 to 700 ms, thus making the heart more susceptible to ventricular arrhythmias and even SCD. One case report of a Chinese child with TS has so far been reported.^[6]^ We aim to study the occurrence of LQTS in Chinese infants and children with syndactyly and will try to identify TS among them.

## 2 Methods

### 2.1 Study design and patients

We study infants and children with syndactyly retrospectively. All those visited the Department of Hand Surgery and Pediatric Orthopedics of Beijing Jishuitan Hospital between July 2003 and February 2013. This study is approved by the Ethics Committee of Beijing Jishuitan Hospital. The need for individual consent is waived by the committee because of the retrospective nature of the study.

Syndactyly is defined as a fusion of adjacent limbs, being fingers or toes. It is classified as ‘simple syndactyly’ when osseous are connected by only skin and soft tissue, and “complex syndactyly” when osseous or cartilaginous unions are present between adjacent digits.^[7]^

The inclusion criteria are:

1)  < 15 years of age; and
2) with complete clinical and examination data, including electrocardiography (ECG), echocardiogram, and blood biochemistry tests. Subjects taking medication that is known to affect the QTc interval or leads to electrolyte disturbance (eg, hypocalcemia, hypokalemia, or hypomagnesemia) are excluded.

### 2.2 Data collection

The demographics, clinical presentation, ECG results, and genetic results have been extracted from the medical charts of all children. In patients with prolonged QTc interval, gene screening has been performed routinely using high-resolution melting and, subsequently, direct sequencing.^[8]^ Exon 8 of the *CACNA1C* gene has been tested in all children, as per local routine practice.^[6]^

### 2.3 ECG

Resting ECG parameters are acquired at the time of admission to the hospital. According to the guidelines,^[9]^ RR intervals and QT intervals in ms in lead II from 5 nonconsecutive beats have been measured, and the corrected QT interval is calculated by dividing the QT interval by the square root of the RR interval using Bazett's formula. The mean values of 3 consecutive QTc have been used. All measurements are completed independently by 2 observers (an attending physician and a cardioelectrophysiologist). In cases of discrepancy, a senior physician has been consulted, and an agreement is reached by consensus. A QTc interval > 450 ms is considered to be abnormally prolonged in children.^[10,11]^

### 2.4 Follow-up

The patients with prolonged QT interval undergo routine ECG and echocardiography yearly. Follow-up was censored on September 30th, 2019.

### 2.5 Statistical analysis

The patients with QTc prolongation are manually matched 1:4 with patients without QTc prolongation according to age and sex. Categorical data are presented as numbers and percentages and are analyzed using Fisher exact test. Continuous variables are presented as means ± standard deviation and are analyzed using the Student *t*-test. Variables that are significantly associated with QTc prolongation are entered into a backward multivariable logistic model. All statistical analyses are performed using SPSS 17.0 (SPSS Inc., Chicago, IL). Two-tailed *P*-values < .05 are considered statistically significant.

## 3 Results

### 3.1 Characteristics of the patients

A total of 355 patients with syndactyly (123 females, 34.6%) have been identified. Seventeen patients (4.8%) show a prolonged QT interval (450–480 ms). The patients with QTc prolongation are manually matched as closely as possible to a 1:4 ratio, and their characteristics are presented in Table 1.

**Table 1:**
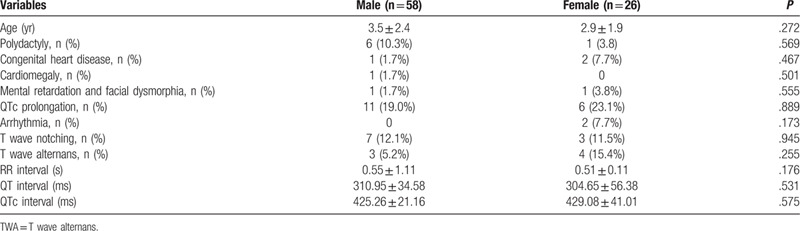
Characteristics of the patients with syndactyly (after matching).

The mean age at admission is 3.4 ± 2.3 years. Of our 255 subjects under study, 7 have polydactyly (8.3%), and 2 mental retardations and facial dysmorphia (2%). Congenital heart disease (CHD) is present in 3 subjects (4%), and various ECG anomalies are seen in 21 subjects (25%): one patient has an atrial septal defect (ASD) with a QTc of 462 ms and special phenotypic features, including ocular hypertelorism and cleft palate; 1 patient (1.2%) has cardiomegaly with a prolonged QTc interval (475 ms); 2 (2.4%) children have arrhythmia; 11 (11.9%) children have T wave notching; and T wave alternans is present in seven children (8.3%). The mean QTc value is 426.4 ± 28.6 ms. Sequencing of exon 8 of the *CACNA1C* gene has been performed from the peripheral leukocytes of the 17 children with prolonged QTc interval. One child has a *CACNA1C* mutation that is already reported.^[6]^ The child with TS is a girl and has been diagnosed at the age of 2 years. She has a 2:1 atrioventricular block and short-rate ventricular tachycardia. She is taking mexiletine, and she has no implanted automatic conversion defibrillator. At the last follow-up, the patient is still alive, without complications, and is 10 years old.

### 3.2 Comparison between children with and without QTc prolongation

As shown in Table 2, compared with the normal QTc group, those with QTc prolongation show higher frequencies of CHD (11.8% vs 1.5%, *P* = .042), mental retardation and facial dysmorphia (11.8% vs 0, *P* = .004), and T wave alternans (23.5% vs 4.4%, *P* = .01).

**Table 2:**
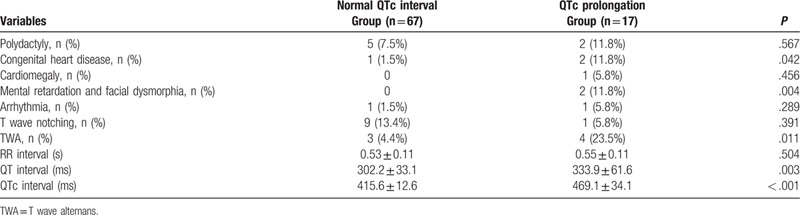
Clinical manifestation and ECG characteristics of the children with syndactyly with or without QTc prolongation.

### 3.3 Multivariable analysis

The univariable analyses show that CHD (*P* = .042), cardiomegaly (*P* = .046), mental retardation and facial dysmorphia (*P* = .004), and T wave alternans (*P* = .01) are associated with QTc prolongation in patients with syndactyly. In the multivariable analysis, only T wave alternans (OR = 10.61, 95%CI: 1.39–81.16, *P* = .023) is independently associated with QTc prolongation in patients with syndactyly (Table 3).

**Table 3:**
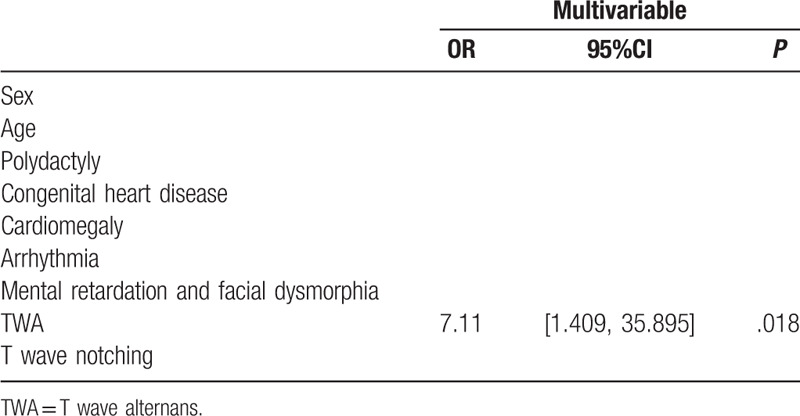
Multivariable logistic regression analysis.

## 4 Discussion

Syndactyly is the most common congenital hand deformity.^[2]^ QTc prolongation is of clinical significance in different pediatric populations such as in children with hypertrophic cardiomyopathy^[12]^ or undergoing CHD surgery.^[13]^ A prolonged QTc interval is an independent risk factor for SCD,^[14,15]^ which is the hallmark of TS.^[16]^ There are little data on children with syndactyly and QTc prolongation. Therefore, this study aims to evaluate the characteristics and long-term outcomes in children with syndactyly and to attempt to identify TS in patients with syndactyly and QTc prolongation. The results suggest that children with syndactyly and prolonged QTc interval have multisystem diseases and ECG abnormalities. T wave alternans is independently associated with QTc prolongation in patients with syndactyly.

In the present study, 5.1% (17/355) of patients with syndactyly also have QTc prolongation. Nuzzi et al^[17]^ demonstrated that the prevalence of prolonged QTc interval is only 0.8% (1/128) among syndactyly patients, which is lower than in the present study. The present study also suggests that the occurrence of T wave alternans is higher in children with prolonged QTc interval than in the normal QTc group. Previous studies showed that T wave alternans reflects ventricular repolarization due to abnormal intracellular Ca^2+^ cycling that may lead to ventricular arrhythmias and could be used as a strong marker of susceptibility to SCD,^[18,19]^ which might explain why children with QTc prolongation are susceptible to SCD.

Consistent with the study by Nuzzi et al,^[17]^ the present study also showed that the patients with prolonged QT intervals do not meet the threshold for TS, but 1 patient is nevertheless identified as having a *CACNA1C* mutation. Several reasons may explain these results. First, because we only identify the classic TS missense mutation p.Gly406Arg, other mutations might have been missed since only 59% of patients with TS have the classic *CACNA1C* mutation.^[5]^ Further study may be needed to examine whether there are other mutations (eg, p.Ser405Arg, p.Gly402Arg, and p.Cys1021Arg) in children with syndactyly with a TS-like phenotype. Second, due to the malignant course of TS with a reported average age at death of 2.5 years,^[20]^ TS patients may not survive to undergo syndactyly-release surgery. Nevertheless, the confirmed case of TS reported here is 10 years old. Another explanation is the possible presence of somatic mosaicism because previous studies report mosaic patients with TS.^[21,22]^ The mutant Ca_v_1.2 channel might be present in probably affected organs (heart and limbs) and be absent from lymphocytes, and samples are not available from tissues characteristically affected by TS (ie, brain and digits).^[16]^ Owning to an extremely low proportion of patients with syndactyly also having TS, preoperative ECG screening combined with genetic testing might be helpful to identify TS among children with syndactyly.

In the present study, all patients have undergone syndactyly release surgery successfully. Nevertheless, patients with prolonged QTc intervals might have a higher risk of fatal arrhythmia when undergoing surgery because many anesthetic agents have the potential to prolong the QT interval, especially when combined with other drugs or electrolyte disturbance.^[23,24]^ In the present study, cardiac events did not occur during anesthesia and surgery, including the patient with TS. Nuzzi et al.^[17]^ report that there are also no cardiac events among 128 patients with syndactyly, including 1 patient with prolonged QT. On the other hand, An et al^[25]^ report 1 patient with syndactyly and TS who suffered from SCD during anesthesia. Walsh et al^[8]^ report that 5 out of 6 patients with TS have a documented cardiac arrest, including 3 occurring during anesthesia and 2 during hospitalization.

QTc prolongation is an independent risk factor for cardiovascular mortality in adult patients,^[26]^ but few data are available concerning the outcome of pediatric patients with QTc prolongation, especially with syndactyly. Anderson et al^[27]^ report that only 1 (1.5%) pediatric death (non-cardiac) did occur in children with isolated QTc prolongation during a 1-year follow-up. Similarly, the present study shows that there are no cardiovascular events in children with syndactyly and QTc prolongation. Punn et al.^[13]^ report that although 44% of children have QTc prolongation post-operatively, it is not associated with adverse mortality, suggesting that children with isolated QTc prolongation might have very low mortality and good prognosis. Nevertheless, such condition might not be suitable for children with LQTS as increased QTc interval is associated with an increased risk of torsades de pointes in this population.^[28]^ Dufendach et al^[5]^ report that 23% of patients die due to ventricular fibrillation and 76% of patients are treated with an implantable cardioverter-defibrillator during a 4.9 years follow-up in patients with TS. implantable cardioverter-defibrillators remain an option for patients with TS, especially for those with refractory arrhythmias.^[5,8]^ In the present study, all patients with QT prolongation are followed by phone and are alive in October 2019, including the patients with confirmed TS, who is 10 years of age.

There are some limitations to this study. First, this is a retrospective study with a relatively small population, which limits the identification of TS because TS is indeed a rare disorder within the general population. Second, the reported prevalence of syndactyly children might be underestimated because of the exclusion of patients with incomplete ECG data. Third, it is unknown whether patients with syndactyly but without QTc prolongation have a positive genetic mutation for TS as genetic testing is not performed in this population. Nevertheless, Kosaki et al^[16]^ report 1 child with syndactyly and a *CACNA1C* mutation not having QT prolongation. Finally, the overall incidence of LQTS is 1:2000 to 1:5000,^[29]^ where the incidence of TS is even lower. Not only does China have no TS incidence rate report, but only 1 patient is reported in China.^[6]^ Unfortunately, the incidence of TS cannot be calculated because we have selected the subjects.

## 5 Conclusions

The results strongly suggest that Chinese children with syndactyly and prolonged QTc interval have multisystem diseases and ECG abnormalities. T wave alternans is independently associated with QTc prolongation in patients with syndactyly.

## Author contributions

**DJZ:** Conceptualization, Funding acquisition, Methodology, Project administration, Supervision, Validation.

**HH:** Conceptualization, Data curation, Investigation, Methodology, Project administration, Resources, Supervision, Roles/Writing - original draft, Writing - review & editing.

**LSN:** Data curation, Investigation, Methodology, Supervision, Validation.

**LYZ:** Formal analysis, Software, Roles/Writing - original draft.

**RL:** Formal analysis, Software, Roles/Writing - original draft, Writing - review & editing.

**SHY:** Formal analysis, Resources.

**ZJQ:** Data curation, Investigation, Methodology.

**ZXS:** Conceptualization, Resources.
